# Progress in clinical genetics of prostate cancer

**DOI:** 10.1186/1897-4287-9-S2-A6

**Published:** 2011-06-01

**Authors:** C Cybulski, D Wokołorczyk

**Affiliations:** 1 Department of Genetics and Pathology, International Hereditary Cancer Center, Pomeranian Medical University, Szczecin, Poland

## 

Prostate cancer is among the leading causes of morbidity and mortality from cancer in men. Epidemiologic data suggest that dominant susceptibility genes may be responsible for 5%-10% of all of the cases and 30-40% of early onset cases. Familial clustering of prostate cancer is observed in 10-20% of men with prostate cancer. Scandinavian study of twins suggests that the heritability of prostate cancer may be as high as 42%. The genetic basis of prostate cancer is complex and appears to involve multiple susceptibility genes. Three candidate susceptibility genes have been positionally cloned—*HPC1*, *HPC2/ELAC2*, and *MSR1*—but a clear role for any of these genes in hereditary prostate cancer has not been established. There is evidence that mutations in *BRCA1* or *BRCA2* predispose to prostate cancer, but the contribution of these two genes to prostate cancer etiology is relatively small. It has been reported that mutations in *NBS1* and *CHEK2* may confer moderate increase in the risk of prostate cancer. We found that germline mutations in *BRCA1*, *CHEK2*, *NBS1* confer increased prostate cancer risk in Polish men. Numerous common low-risk polymorphisms associated with prostate cancer have been reported, many located in genes involved in the DNA damage repair and cell cycle control pathways (*i.e. CDKN1B*, *BRCA2*, *ATM*, *XRCC1*, *XRCC2*, *ERCC2*), however most of these associations have not been replicated. In the past two years, the results of several genome-wide searches for prostate cancer susceptibility loci have been reported. Several chromosomal regions of interest have been identified, including loci on chromosomes 2, 3, 6, 7, 8, 10, 11, 17, 19 and X. It is believed that identification of genetic markers for prostate cancer will improve prevention, diagnosis and management with prostate cancer.

